# The relation between residual stress, interfacial structure and the joint property in the SiO_2f_/SiO_2_-Nb joints

**DOI:** 10.1038/s41598-017-04531-w

**Published:** 2017-06-23

**Authors:** Qiang Ma, Zhuo Ran Li, Lai Shan Yang, Jing Huang Lin, Jin Ba, Ze Yu Wang, Jun Lei Qi, Ji Cai Feng

**Affiliations:** State Key Laboratory of Advanced Welding and Joining, Harbin Institute of Technology, Harbin, 150001 China

## Abstract

In order to achieve a high-quality joint between SiO_2f_/SiO_2_ and metals, it is necessary to address the poor wettability of SiO_2f_/SiO_2_ and the high residual stress in SiO_2f_/SiO_2_-Nb joint. Here, we simultaneously realize good wettability and low residual stress in SiO_2f_/SiO_2_-Nb joint by combined method of HF etching treatment and Finite Element Analysis (FEA). After etching treatment, the wettability of E-SiO_2f_/SiO_2_ was improved, and the residual stress in the joint was decreased. In order to better control the quality of joints, efforts were made to understand the relationship between surface structure of E-SiO_2f_/SiO_2_ and residual stress in joint using FEA. Based on the direction of FEA results, a relationship between residual stress, surface structure and joint property in the brazed joints were investigated by experiments. As well the FEA and the brazing test results both realized the high-quality joint of E-SiO_2f_/SiO_2_-Nb and the shear strength of the joint reached 61.9 MPa.

## Introduction

SiO_2f_/SiO_2_, one of the most significant functional and structural quartz fiber reinforced silica ceramic matrix composites, has been attracted great attention in aerospace industry applications, due to its high thermal shock resistance, excellent ablation resistance as well as low thermal conductivity^1–6^. However, like most advanced ceramic matrix composites, it is difficult for SiO_2f_/SiO_2_ to fabricate large-sized or complex-shaped components owing to its intrinsic brittleness and low fracture strain^7–12^. Therefore, it is indispensable to develop a reliable technique to joint SiO_2f_/SiO_2_ with metals to extend the application of the composite.

Due to the intrinsic brittleness of SiO_2f_/SiO_2_, usually, fusion welding is not applied because of the possibility of brittle fracture forming during cooling^13^. Alternatively, although adhesive bonding can be performed to realize SiO_2f_/SiO_2_-metal joint, the strength of the joint reduce during long service. Consequently, vacuum brazing is the primary method to joining SiO_2f_/SiO_2_ to metals because of its cost-effectiveness and high-quality process^14–22^. However, two crucial challenges: the poor wettability of SiO_2f_/SiO_2_ and the high residual stress in the joint exist in brazing SiO_2f_/SiO_2_ to metal, which seriously impairs the strength of the joints. In view of that, some researchers demonstrated that the wettability of SiO_2f_/SiO_2_ with active brazing alloy can be successfully improved by coated nickel^23^, CaCO_3_^24^, or few-layer graphene^25^. Although the wettability of SiO_2f_/SiO_2_ with active brazing alloy is improved, a high-quality joint is difficult to obtain because of the negligence to the high residual stress.

So far, by micro-machine process on the surface of ceramic or composite, forming a 3D composite-metal gradient transition zone in their joints has been developed to reduce the residual stress of the brazed joints^26–28^. Zhang *et al*.^26^ demonstrated that fabricating microscale periodic surface pattern on Al_2_O_3_ ceramic surface was a promising method to form a 3D transition region and reduce the residual stress of ceramic-metal joints. Wang *et al*.^27^ reported that drilling holes on the surface of C/C composite can reduce the residual stress because of constructing a 3D C/C-metal gradient transition zone in joint. Shen *et al*.^28^ demonstrated that drilling blind holes on C/C composite surface by means of laser can result in significantly strengthening and toughening the joint due to creating a 3D transition region between the C/C and braze. To some extent, micro-machining on its surface can lead to damaging the whole structure of SiO_2f_/SiO_2_, due to its intrinsic brittleness and braided structure. Undoubtedly, it is urgently to provide an effective method to both improving the wettability with active brazing alloy and forming a 3D gradient transition zone at SiO_2f_/SiO_2_ side without impairing the overall structure. Our latest work showed that a 3D-pinning structure was beneficial to the joint strength. However, the structure introduced the residual stress among the braided quartz fibers, which complicated the distribution of the residual stress in the joint. Furthermore, the complex structure of the 3D SiO_2f_/SiO_2_-metal gradient transition zone had a decisive influence on the mechanical property of joint. Thus, the relationship between the residual stress, the surface structure and joint property in the brazed joints need be explored in depth and in detail.

In this paper, the etching treatment with HF acid solution was designed to regulate the surface structure of SiO_2f_/SiO_2_. Finite element analysis (FEA) models were developed to study the relationship between the residual stress and the surface structure in the brazed joints, which can guide the subsequent experiment and provide the theoretical basis. Furthermore, the relationship between the residual stress, the surface structure and the joint property in the brazed joints was discussed in detail.

## Experimental procedures

### Materials

The 3D four-directional braided SiO_2f_/SiO_2_^29^ and commercially available Nb were used as the parent materials. The dimension of SiO_2f_/SiO_2_ brazing specimen was 5 mm × 5 mm × 3 mm. Nb was cut into 10 mm × 10 mm × 3  mm slices for the microstructure observation and 10 mm × 15 mm × 3 mm for shear tests, respectively. The active brazing alloy foil Ag-21Cu-4.5Ti (wt.%) with a thickness of 200 μm was used to braze SiO_2f_/SiO_2_ and Nb. The bonding surfaces of samples were ground up to 400 by SiC sandpaper. All materials were ultrasonically cleaned in acetone for 15 min.

**Figure 1:**
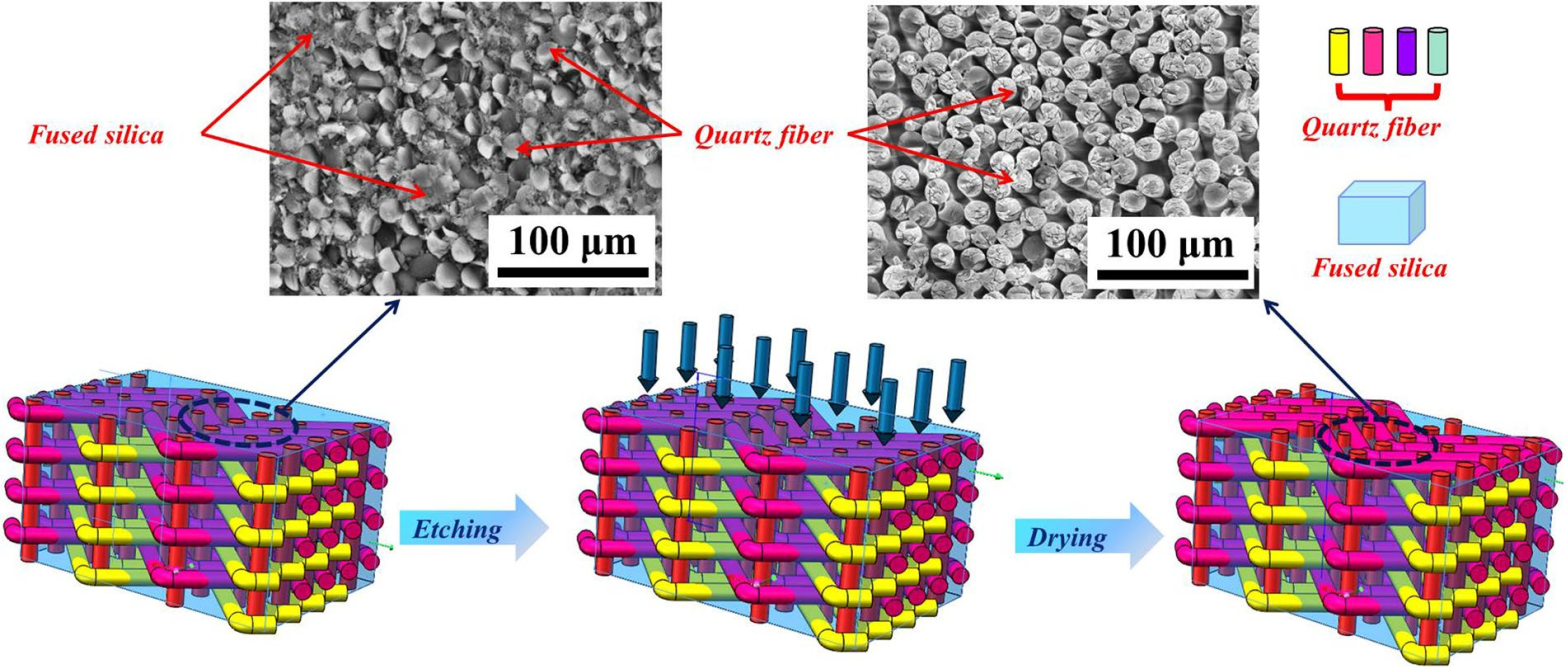
Schematic of etching treatment on SiO_2f_/SiO_2_.

The etching treatment on the surface of SiO_2f_/SiO_2_ was performed with 20 wt.% HF acid solution. The schematic of the etching process is shown in Fig. 1. Firstly, the HF acid solution was directly placed on the surface of SiO_2f_/SiO_2_ for a few seconds. Then, the surface was washed with deionized water carefully. Finally, SiO_2f_/SiO_2_ with etching treatment (E-SiO_2f_/SiO_2_) was obtained. In addition, by controlling etching process, the surface structure dimension can be tunable.

### Wetting and brazing processes

Wetting experiments were performed using the sessile drop technique in which the alloys are placed on the substrate and the system is heated to 840 °C. For comparison, during the sessile drop experiments, AgCuTi brazing alloy foils were placed on SiO_2f_/SiO_2_ with different infiltration depth, respectively. And the brazing experiments were performed with AgCuTi active brazing alloy foils between the parent materials. The structure of the assembly was SiO_2f_/SiO_2_/AgCuTi/Nb, and the assemblies were held by graphite jigs. In order to keep the specimens in close contact, a load of 0.01 MPa was applied. The assemblies were heated to 840 °C with a rate of 10 °C min^−1^ in a vacuum furnace, isothermally held for 10 min, and then cooled down to room temperature at a rate of 5 °C min^−1^.

The drop images, which were produced by an optical system coupled with a zoom (magnification 30×), were recorded by a video camera connected to a computer, permitting automatic image analysis. This device enables the contact angles of the drop were measured with an accuracy of ±2°. The interfacial microstructures of the joints were analyzed by a scanning electron microscopy (SEM) fitted with an energy dispersive spectroscopy (EDS). To identify the phases formed in the reaction layers adjacent to SiO_2f_/SiO_2_ and fracture, a JDX-3530M X-ray diffraction (XRD) was used. To evaluate the mechanical properties of the joints, shear tests were carried out using an Instron-1186 universal testing machine at room temperature. The average stress strength was identified by five shear specimens brazed under the same condition.

### FEA calculations

The FEA method was employed to investigate the distribution of the residual stress along the SiO_2f_/SiO_2_-Nb brazed joint in our research, because the method was proved to be a useful tool for predicting residual stress in the joint^30, 31^. Thus, in this paper, the distribution of the residual stress, which yielded in the joint during cooling due to the mismatch of Coefficient Thermal Expansion (CTE), was simulated by FEA with Marc-2013.

## Results and Discussion

### Microstructure of the joint brazed with and without etching treatment

**Figure 2:**
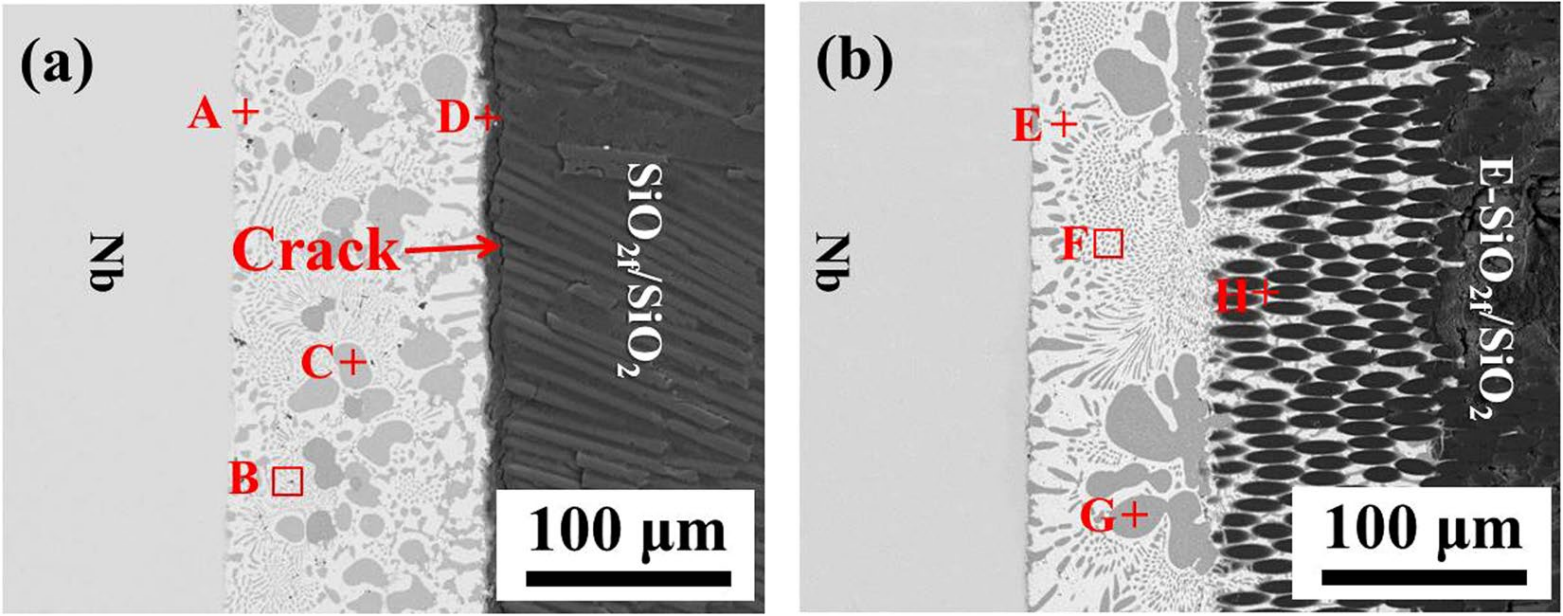
SEM images of (**a**) SiO_2f_/SiO_2_-Nb and (**b**) E-SiO_2f_/SiO_2_-Nb joint.

The interface analysis was first performed on the brazing joints before and after etching treatment, evidencing the establishment and the application of model for FEA. The typical microstructure of SiO_2f_/SiO_2_-Nb joint is shown in Fig. 2a. It can be found cracks formed at SiO_2f_/SiO_2_ side, which the typical interface structure of the joint was SiO_2f_/SiO_2_/Ag(s,s) + Cu(s,s)/Cu_3_Ti_3_O/TiSi_2_, based on our latest research. And the mismatch of CTE between SiO_2f_/SiO_2_ (CTE_SiO2f/SiO2_ = ~2.0 × 10^−6^/K) and Nb (CTE_Nb_ = ~7 × 10^−6^/K) or AgCuTi active brazing alloy (CTE_AgCuTi_ = ~15.4 × 10^−6^/K) is high, which results in forming cracks^32^. Figure 2b shows the typical microstructure of E-SiO_2f_/SiO_2_-Nb joint. Compared with SiO_2f_/SiO_2_-Nb joint, the primary compositions of beam were nearly same. The brazing alloy infiltrated into E-SiO_2f_/SiO_2_ and formed a “3D-pinning structure”, which contributed to form a good CTE gradient transition and to reduce its mismatch between different materials^27, 33^. Consequently, the joint exhibited sound bonding without any defect and crack.

**Figure 3:**
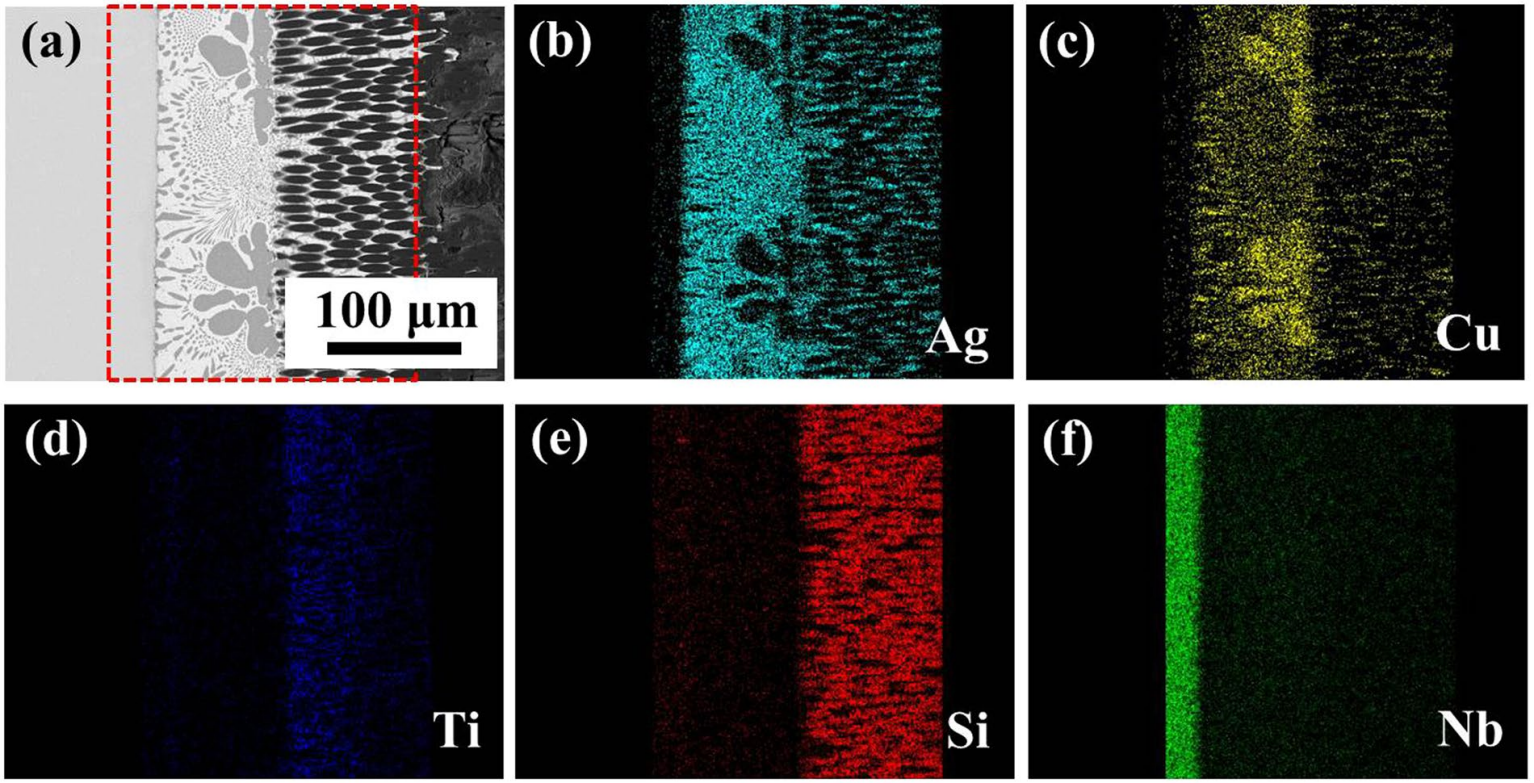
Interfacial microstructure and elemental distribution of E-SiO_2f_/SiO_2_-Nb joint (**a**) BSE image of the joint and EDS maps of (**b**) Ag, (**c**) Cu, (**d**) Ti, (**e**) Si and (**f**) Nb.

**Figure 4:**
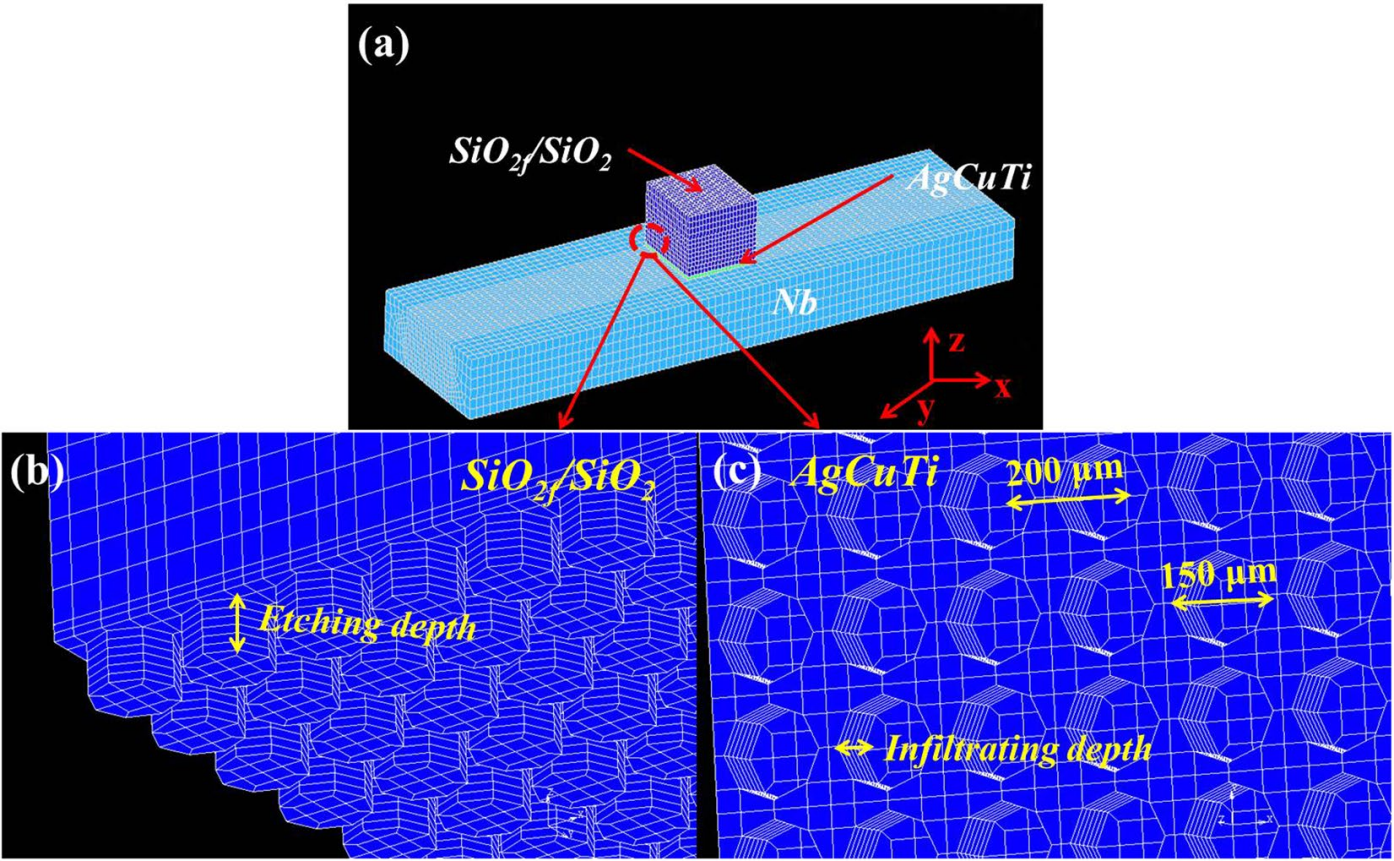
Mesh of finite elements for (**a**) the brazed joint, (**b**) magnification of the E-SiO_2f_/SiO_2_, (**c**) magnification of AgCuTi brazing alloy.

In order to further investigate the interfacial microstructure of the E-SiO_2f_/SiO_2_-Nb joint, the main elements distribution of the joint produced at 840 °C for 10 min are analyzed, as shown in Fig. 3. The Fig. 3a clearly presents that a sound joint has been obtained. Figure 3b–f shows the distribution of Ag, Cu, Ti, Si and Nb, respectively. It can be seen that the Si had a strong tendency to extremely react with Ti, as shown in Fig. 3d and e. In addition, notice that Ti-rich granular were formed adjacent to SiO_2f_/SiO_2_ composite, revealed that Ti segregated in 3D SiO_2f_/SiO_2_-metal gradient transition zone. Furthermore, the distribution of Ti in that zone was not even because the brazing alloy gradually infiltrated into E-SiO_2f_/SiO_2_, and Ti reacted with the contacted quartz fibers. Thus, the remaining Ti became less and less as the infiltration depth increasing. Moreover, it was important to note that SiO_2f_/SiO_2_ and Nb did not spread or dissolve during brazing, as shown in Fig. 3b and f, respectively. Therefore, based on the above results, the model for FEA can be developed as three parts: SiO_2f_/SiO_2_ (or E-SiO_2f_/SiO_2_), AgCuTi brazing alloy and Nb (see Fig. 4). It was worth noting that the special structure of the E-SiO_2f_/SiO_2_ by the etching treatment needed a completely new design system (Details on the model were shown in supplementary material).

### Estimation of residual stress in the brazed joint using FEA

Recently, many researches have focused on reducing the residual stress in the composite/ceramic and metal brazed joints^34–37^. In fact, it is very difficult to measure the residual stress of the brazed joints directly through the experimental measurement. Thus, it is general to analyze the residual stress of the brazed joints from the joint fracture path. However, this analysis only horizontal contrast (trend), cannot be quantified (experimental value) contrast. In addition, when the fracture path exists in the same area, it is difficult to analyze the residual stress of composite/ceramic and metal brazed joints. In order to analyze the residual stress variation better, some researchers always investigated the distribution of residual stress by Finite Element Analysis (FEA)^38, 39^. In our case, the fracture path is different before and after etching treatment^40^. Especially, after etching treatment, fractures all exist close to the etching area, then it is not accurate to analyze the residual stress through the fracture path. Therefore, based on the typical experimental results, the values of residual stress of the samples with varied etching depth can be examined by FEA method.

Based on the analysis in *3.1* section, the FEA was applied to simulate the distribution of residual stress in the brazed joint, and the details on simulation process were shown in supplementary material. The etching depth was related to the dimension of “3D-pinning structure” which directly affected the residual stress in the joint. Therefore, it is important to investigate the relationship between the surface structure (that is the layer thickness of 3D-pinning structure) and the residual stress in a brazed joint, which can provide the theoretical basis for the following brazing experiments.

**Figure 5:**
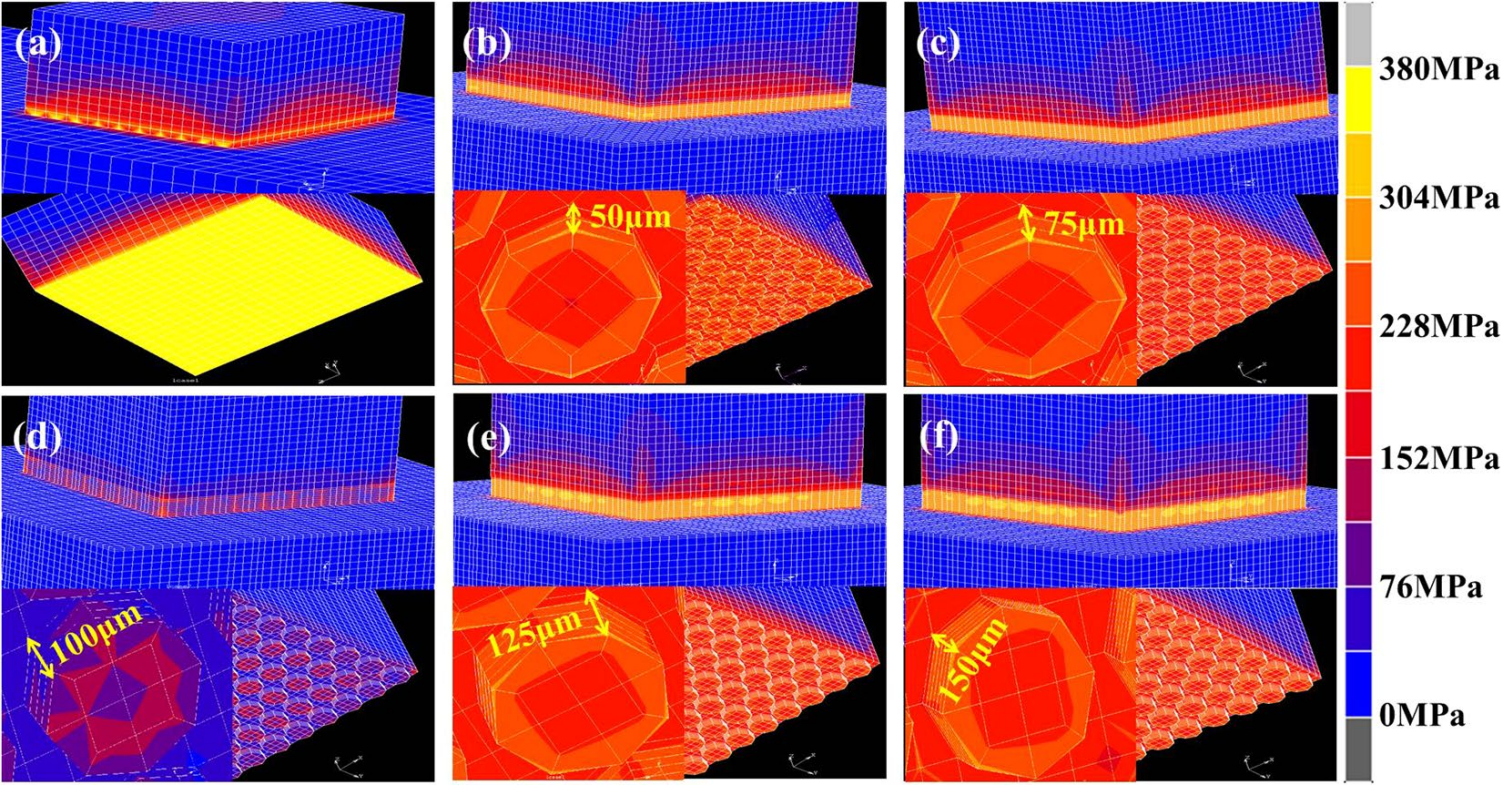
Distribution of equivalent von mises stress in (**a**) 0 μm@E-SiO_2f_/SiO_2_-Nb, (**b**) 50 μm@E-SiO_2f_/SiO_2_-Nb, (**c**) 75 μm@E-SiO_2f_/SiO_2_-Nb, (**d**) 100 μm@E-SiO_2f_/SiO_2_-Nb, (**e**) 125 μm@E-SiO_2f_/SiO_2_-Nb and (**f**) 150 μm@E-SiO_2f_/SiO_2_-Nb joint.

**Figure 6:**
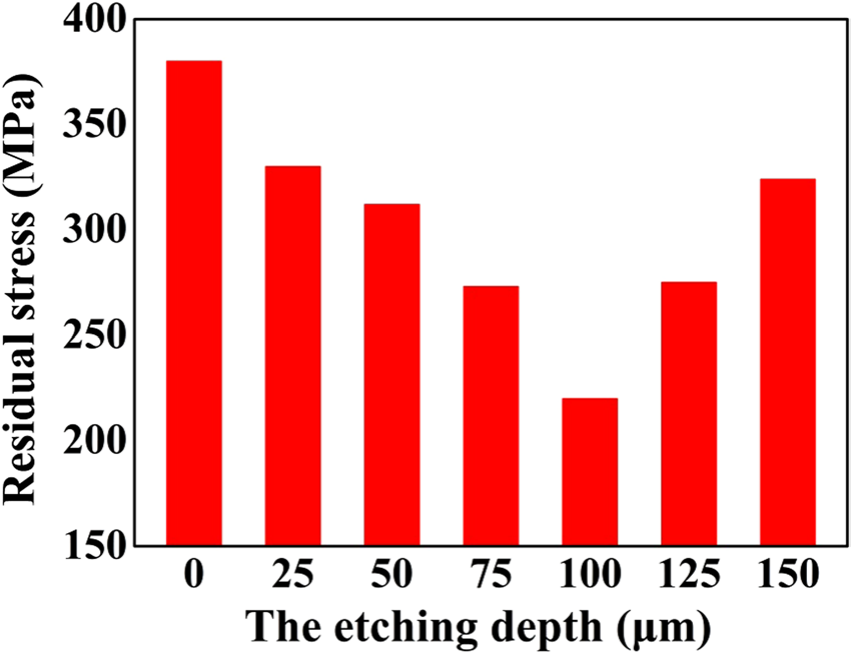
Comparison of equivalent von mises stress for different etching depth.

Figure 5 shows the distribution of equivalent von mises stress in 0 μm@E-SiO_2f_/SiO_2_-Nb (denoted as “Xμm@E-SiO_2f_/SiO_2_-Nb for convenience, X represented the etching depth), 50 μm@E-SiO_2f_/SiO_2_-Nb, 75 μm@E-SiO_2f_/SiO_2_-Nb, 100 μm@E-SiO_2f_/SiO_2_-Nb, 125 μm@E-SiO_2f_/SiO_2_-Nb and 150 μm@E-SiO_2f_/SiO_2_-Nb joints brazed by AgCuTi at 840 °C for 10 min. It is obvious that the residual stress has been gradually changed with the etching depth increasing from 0 μm to 150 μm. As for 0 μm@E-SiO_2f_/SiO_2_-Nb joint (that is SiO_2f_/SiO_2_-Nb joint), the residual stress is constrained around the 0 μm@E-SiO_2f_/SiO_2_-AgCuTi interface, and the peak residual stress of 380 MPa (see Fig. 6) generates in the SiO_2f_/SiO_2_ side close to brazing alloy, and then gradually decreased along the vertical direction of 0 μm@E-SiO_2f_/SiO_2_-AgCuTi interface. After etching treatment, the higher residual stress in E-SiO_2f_/SiO_2_-Nb joints has transferred in the “3D-pinning structure”, which suggested that the structure played a key role in the distribution of the residual stress in the joints (see Fig. 5b–f). Moreover, it is worth noting that the maximum residual stress of E-SiO_2f_/SiO_2_-Nb joints has transferred on the braided quartz fibers in the “3D-pinning structure”, as shown in Fig. 5b–f. Furthermore, it clearly presents that the residual stress in the joints and on the braided quartz fibers were both reduced with the etching depth increasing. However, when the etching depth was too thick, the residual stress increased significantly, especially on the braided quartz fibers, as shown in Fig. 5d–f. From the above results, it can be inferred that the “3D-pinning structure” can effectively reduce the residual stress and change the distribution of residual stress in the joints.

**Figure 7:**
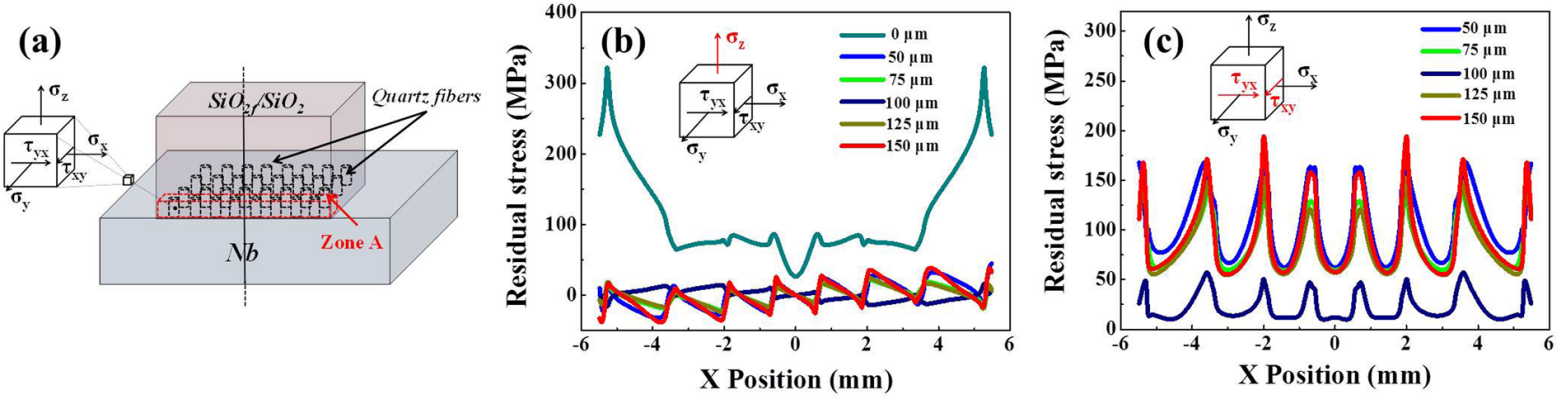
(**a**) Schematic diagram of model and coordinate half system adopted in calculation and residual stress distribution of SiO_2f_/SiO_2_ side closed to interface (**b**) σ_z_, (**c**) τ_xy_.

As for the 0 μm@E-SiO_2f_/SiO_2_-Nb joints, the high CTE mismatch may cause the residual stress constraining around the SiO_2f_/SiO_2_-AgCuTi interface. After etching treatment, a “3D-pinning structure” formed in the joint, which was contributed the brazing alloy infiltrating into the E-SiO_2f_/SiO_2_ side, forming a 3D SiO_2f_/SiO_2_-metal gradient transition zone. The zone was beneficial to reduce the residual stress induced by the high mismatch of the dissimilar substrates. However, the zone also made the distribution of the residual stress in “3D-pinning structure” complicated, as shown in Fig. 5. In particular, when the etching depth further increased, the residual stress rather than reduced. In order to analyses the reason, we explored the relationship between the residual stress of joint in different directions and surface structure of the E-SiO_2f_/SiO_2_ by FEA. The schematic diagram of the profile of the model used in simulation procedure is shown in Fig. 7a. According to the structure of the E-SiO_2f_/SiO_2_-Nb joints and our calculation results, it can be inferred that principal stress σ_z_ changed with the etching depth increasing, but σ_x_ and σ_y_ did not or very little change. Another significant stress was shear stress τ_xy_, which latter, in combination with σ_z_, can induce fracture of the quartz fibers^41^. In addition, shear stress τ_xz_ and τ_zy_ changed very little with the etching depth increasing. So, the following analysis only focused on the largest principal stress σ_z_ and shear stress τ_xy_ in E-SiO_2f_/SiO_2_-Nb joints. Figure 7b and c show the maximal σ_z_ and τ_xy_ in zone A of E-SiO_2f_/SiO_2_-Nb joints, respectively. It can be observed that after etching treatment, σ_z_ reduced markedly (from 0 to 100 μm), but a little varied with the etching depth increasing to 150 μm, as shown in Fig. 7b. In contrast, it is noteworthy that τ_xy_ significantly increased with the etching depth further increasing (from 100 to 150 μm), as shown in Fig. 7c. Thus, the maximal resultant force in the zone descended first (from 0 to 100 μm) and then ascended (from 100 to 150 μm), as shown in Fig. 6. From the above results, it can be concluded that with the etching depth between 0 to 100 μm, the residual stress of the joint can be reduced, because of the formed 3D SiO_2f_/SiO_2_-metal gradient transition zone, which was contributed to decrease the CTE mismatch. Nevertheless, with the etching depth further increasing (from 100 to 150 μm), the residual stress was no lower, but higher, due to the τ_xy_ which was introduced by the zone and posed serious problems for the joint. Therefore, it can be concluded that although the “3D-pinning structure” can reduce the residual stress, the dimension of the structure should be in proper domain.

### Effect of surface structure on the wettability of SiO_2f_/SiO_2_ composite

**Figure 8:**
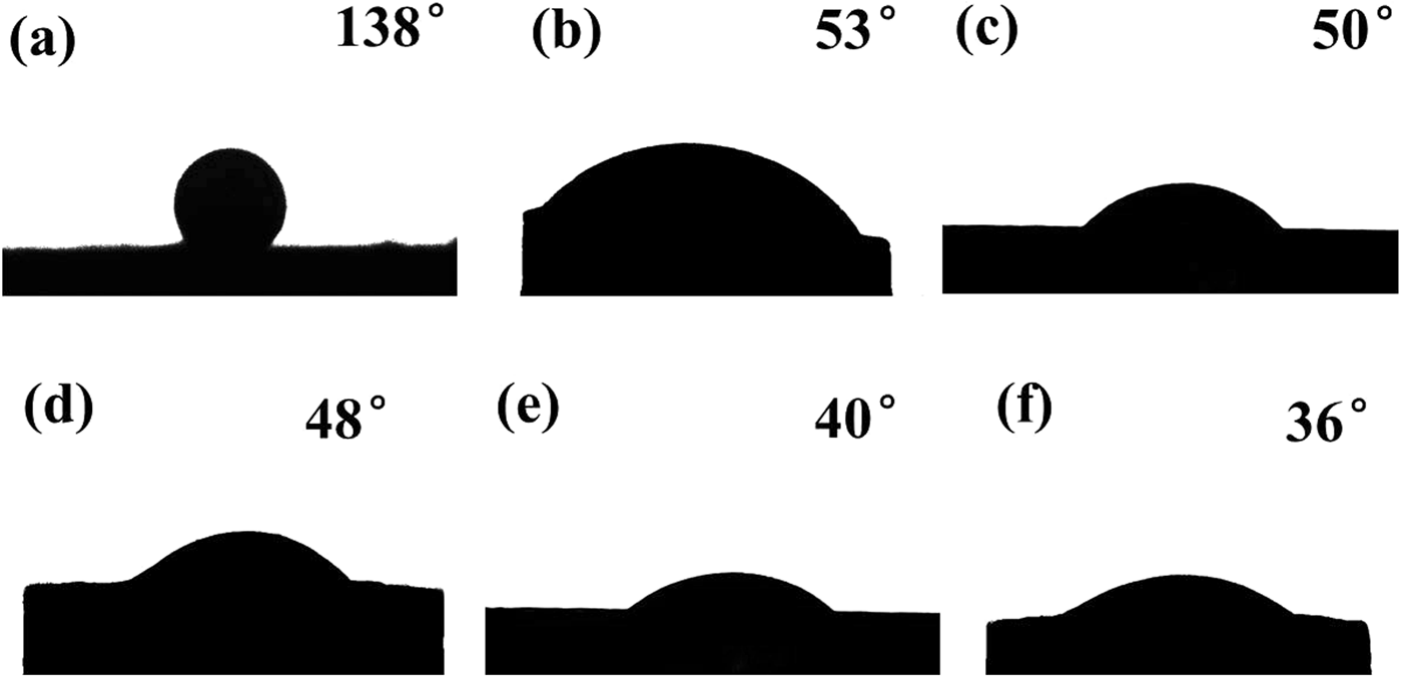
Contact angles of AgCuTi brazing alloy on the (**a**) 0 μm@E-SiO_2f_/SiO_2_, (**b**) 50 μm@E-SiO_2f_/SiO_2_, (**c**) 75 μm@E-SiO_2f_/SiO_2_, (**d**) 100 μm@E-SiO_2f_/SiO_2_, (**e**) 125 μm@E-SiO_2f_/SiO_2_ and (**f**) 150 μm@E-SiO_2f_/SiO_2_.

It is well known that the wettability of SiO_2f_/SiO_2_ plays an important role in obtaining a high-quality brazing joint^42, 43^. Therefore, it is necessary to investigate the effect of etching treatment on the wettability of AgCuTi brazing alloy on SiO_2f_/SiO_2_ surface. Figure 8 shows the contact angles (CA) of AgCuTi brazing alloy on the surface of 0 μm@E-SiO_2f_/SiO_2_, 50 μm@E-SiO_2f_/SiO_2_, 75 μm@E-SiO_2f_/SiO_2_, 100 μm@E-SiO_2f_/SiO_2_, 125 μm@E-SiO_2f_/SiO_2_ and 150 μm@E-SiO_2f_/SiO_2_, respectively. It can been observed that the brazing alloy on SiO_2f_/SiO_2_ shows unsymmetrical round shape, with left side showing smaller contact angles compare to the right one. And there are two main reasons for it. Firstly, the SiO_2f_/SiO_2_ brazing specimen was obtained through cutting into slices and grounding the bonding surface. So, after that treatment, the bonding surface of SiO_2f_/SiO_2_ was not flat, which led to the surface of E-SiO_2f_/SiO_2_ uneven, even the slope. Secondly, the surface stability of liquid brazing alloy on E-SiO_2f_/SiO_2_ was affected in vacuum furnace by mechanical pump and molecular pump during vacuumizing. Therefore, the brazing alloy on the surface of E-SiO_2f_/SiO_2_ showed unsymmetrical round shape in Fig. 8. However, the left contact angles (CA) of AgCuTi brazing alloy on the E-SiO_2f_/SiO_2_ decreased from 134° to 32° and the right CA decreased from 138° to 36°, as shown in Fig. 2. The CA decreased with the etching depth increasing, though the brazing alloy on SiO_2f_/SiO_2_ shows unsymmetrical round shape. Then, in our case, the right CA acted as the evaluation standard.

**Figure 9:**
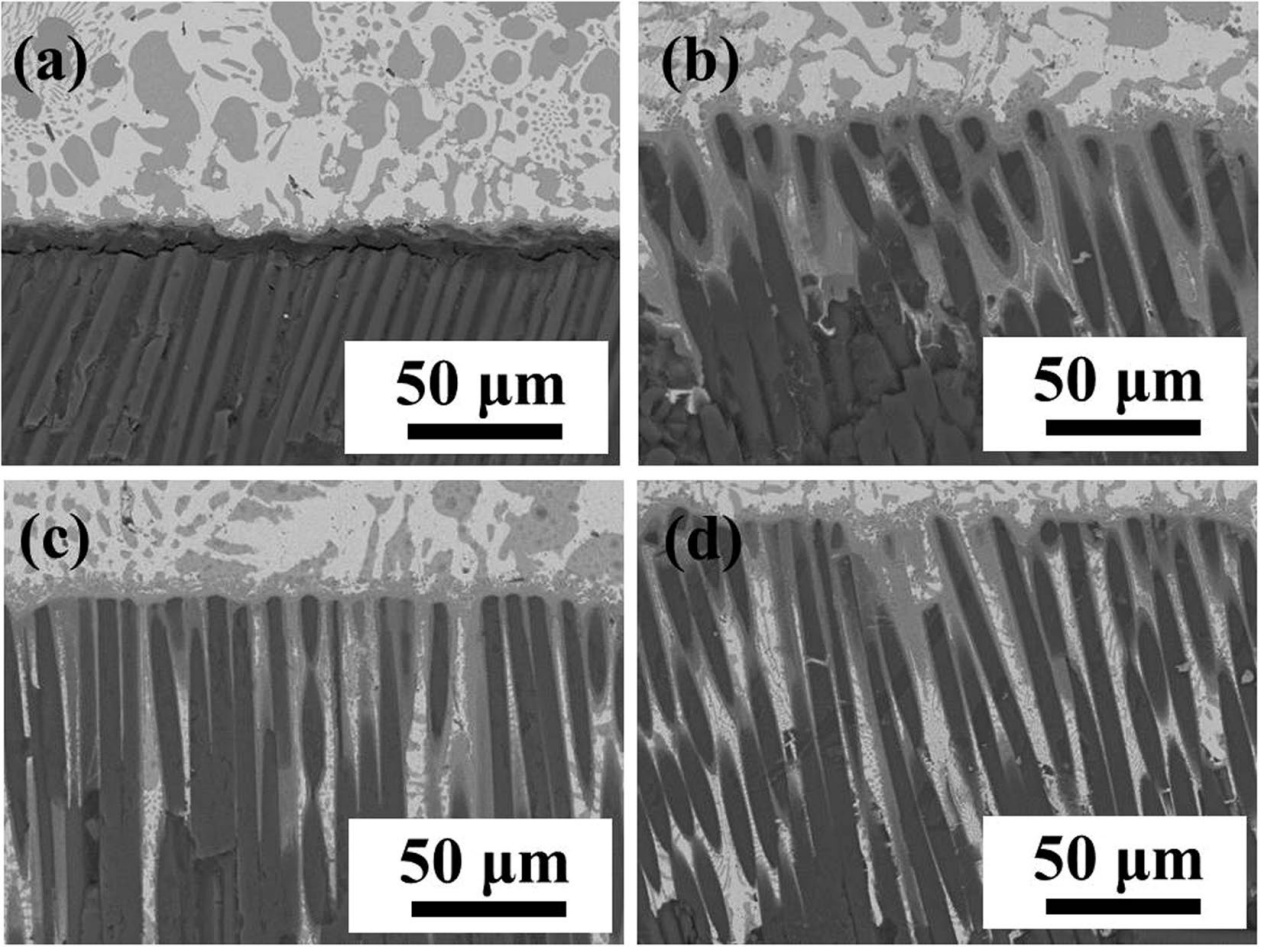
SEM images of wetting region in (**a**) 0 μm@E-SiO_2f_/SiO_2_, (**b**) 50 μm@E-SiO_2f_/SiO_2_, (**c**) 100 μm@E-SiO_2f_/SiO_2_ and (**d**) 150 μm@E-SiO_2f_/SiO_2_.

Figure 8a shows the CA of AgCuTi brazing alloy on the surface of SiO_2f_/SiO_2_ was up to 138°, indicating poor wettability. After etching treatment, Fig. 8b shows the CA of AgCuTi brazing alloy on the 50 μm@E-SiO_2f_/SiO_2_ was significantly decreased to 53°, meaning favorable wettability. Furthermore, it can be seen that the CA decreased from 53° to 36° with the etching depth increasing. As a result, it suggested that after etching treatment, the wettability of all E-SiO_2f_/SiO_2_ was excellent. In order to further explore the reasons for the improving wettability, the microstructures of wetting region were analyzed in details, as shown in Fig. 9. From Fig. 9a, it can be observed the weak joining between SiO_2f_/SiO_2_ and brazing alloy, because of the poor wettability of SiO_2f_/SiO_2_. In contrast, it can be observed that after etching treatment, the brazing alloy infiltrated into E-SiO_2f_/SiO_2_ and sound metallurgical bonding was formed between brazing alloy and braided quartz fibers (see Fig. 9b). Furthermore, the infiltration depth was gradually increased with etching depth increasing, as shown in Fig. 9. After etching treatment, only the silica sol was consumed and numerous quartz fibers were left on the surface of SiO_2f_/SiO_2_^17^. Based on the above results, we believe that the poor wettability of SiO_2f_/SiO_2_ can be owing to the silica sol, and the wettability between brazing alloy and quartz fibers was extremely well. It suggested that the etching treatment was an easy and effective way to improve the wettability of SiO_2f_/SiO_2_. Thus, it can be inferred that the space of the consumed silica sol was able to be filled up with sufficient brazing alloy.

**Figure 10:**
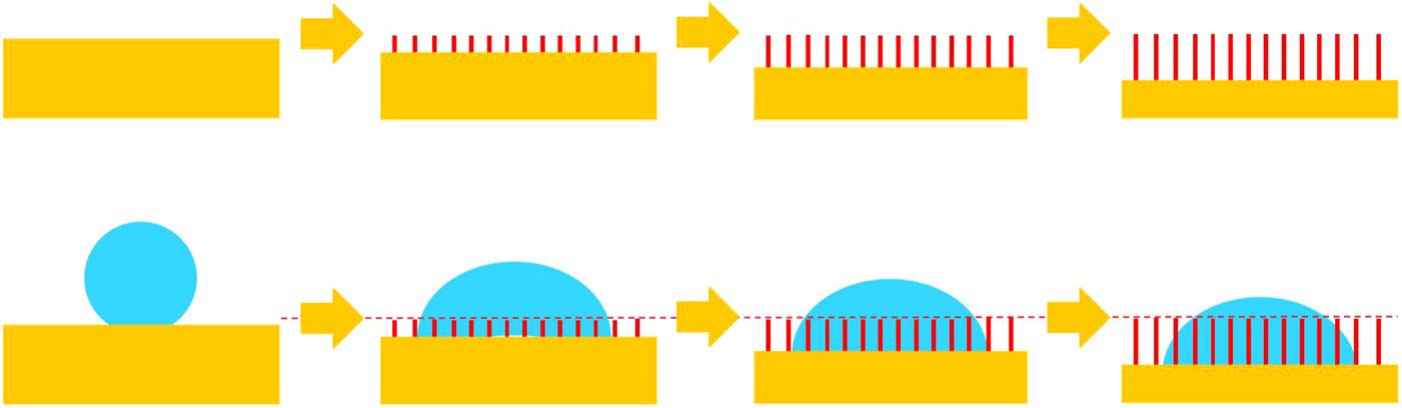
Schematic of wetting evolution process.

Generally, the wettability of the materials with the same surface state is almost identical under the same condition. The surface of SiO_2f_/SiO_2_, after etching treatment, is all the quartz fibers were left, which are the same regardless of the etching depth. However, it is worth noting that the CA of AgCuTi brazing alloy on the surface of E-SiO_2f_/SiO_2_ decreased with the etching depth increasing, as shown in Fig. 8. In order to illustrate the wetting process, a concept physical model was established, as shown in Fig. 10. After etching treatment, the fused silica was consumed and quartz fibers were left in the transition zone, which contributed to the brazing alloy infiltrating into E-SiO_2f_/SiO_2_. Furthermore, the width of the transition zone increased with the etching depth increasing, and then less and less brazing alloy was left on the surface of E-SiO_2f_/SiO_2_, as shown in Fig. 10. Thus, the CA of E-SiO_2f_/SiO_2_ decreased with etching depth increasing from the wetting experiments results. In fact, the CA of E-SiO_2f_/SiO_2_ was constant. According to the above results, it is reasonable to infer that the space of the consumed silica sol can be filled up with brazing alloy, as long as it was enough.

### Effect of surface structure of SiO_2f_/SiO_2_ on the joint microstructure and property

**Figure 11:**
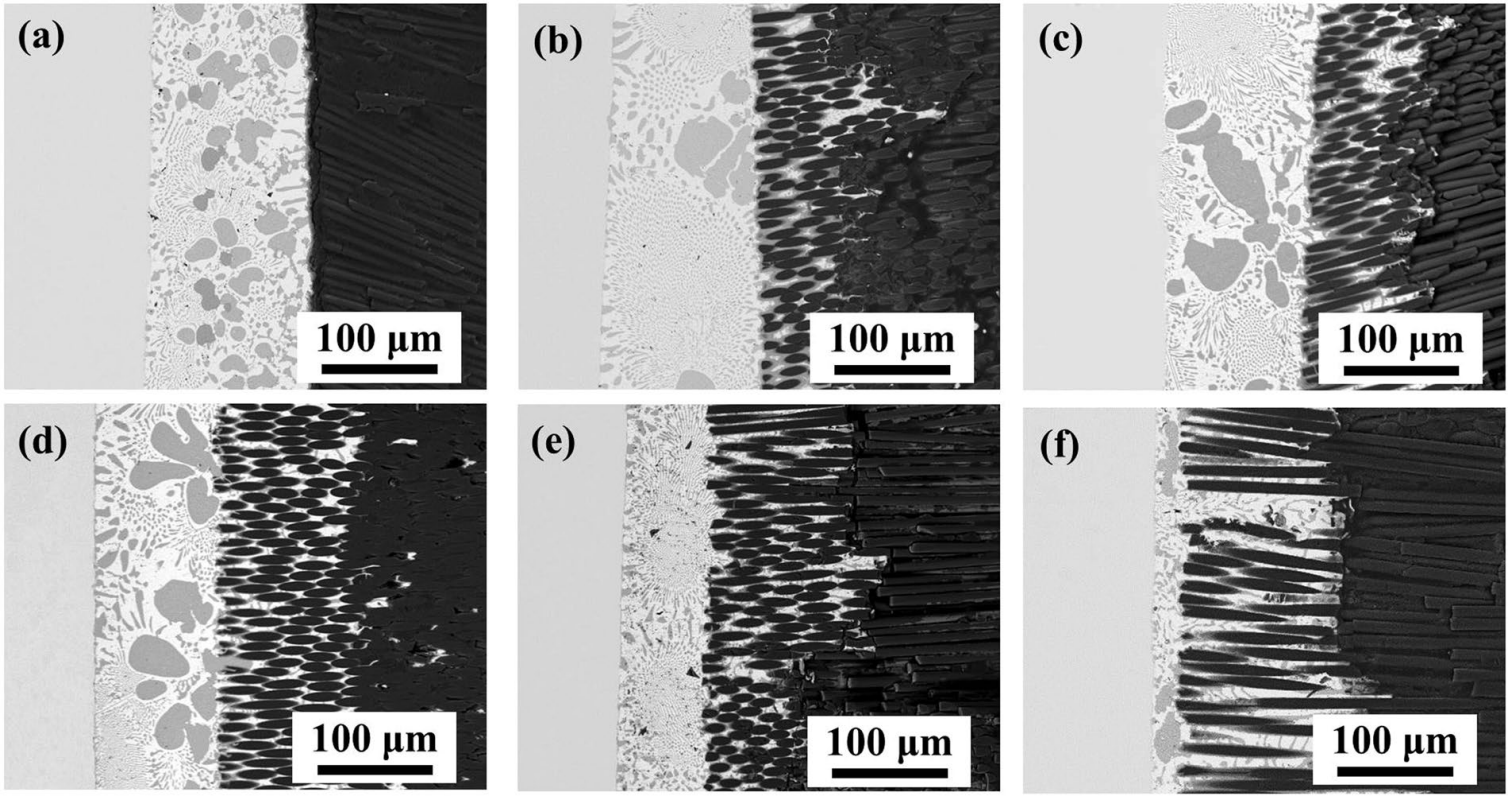
SEM images of (**a**) 0 μm@E-SiO_2f_/SiO_2_-Nb, (**b**) 50 μm@E-SiO_2f_/SiO_2_-Nb, (**c**) 75 μm@E-SiO_2f_/SiO_2_-Nb, (**d**) 100 μm@E-SiO_2f_/SiO_2_-Nb, (**e**) 125 μm@E-SiO_2f_/SiO_2_-Nb and (**f**) 150 μm@E-SiO_2f_/SiO_2_-Nb joint.

**Figure 12:**
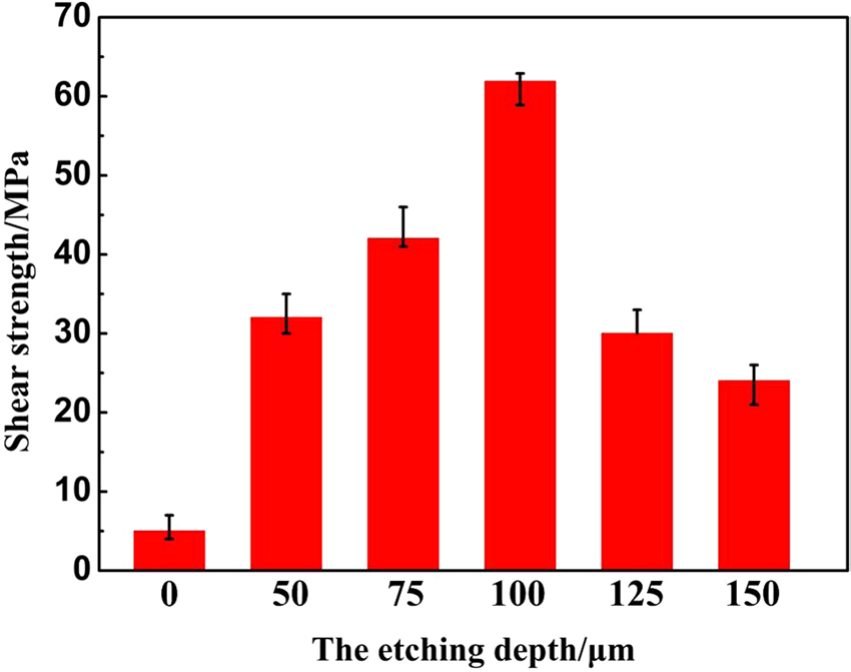
Shear strength of brazed joints with different interfacial structure.

In order to further investigate the relationship between the residual stress, the surface structure and the joint property in the brazed joints, the joining strength and the microstructure evolution was comparatively studied. The typical microstructure of 0 μm@E-SiO_2f_/SiO_2_-Nb, 50 μm@E-SiO_2f_/SiO_2_-Nb, 75 μm@E-SiO_2f_/SiO_2_-Nb, 100 μm@E-SiO_2f_/SiO_2_-Nb, 125 μm@E-SiO_2f_/SiO_2_-Nb and 150 μm@E-SiO_2f_/SiO_2_-Nb joints brazed by AgCuTi brazing alloy at 840 °C for 10 min are shown in Fig. 11. It is obvious that the interfacial microstructure changed with the depth of brazing alloy infiltrating into SiO_2f_/SiO_2_ increasing. As for 0 μm@E-SiO_2f_/SiO_2_-Nb joint, continuous cracks can be observed in SiO_2f_/SiO_2_ side near the brazing interface (see Fig. 11a). It may be because the poor wettability of SiO_2f_/SiO_2_ and the high residual stress induced by the CTE mismatch between SiO_2f_/SiO_2_ and Nb. So, the shear stress of the 0 μm@E-SiO_2f_/SiO_2_-Nb joint was only 5 MPa (see Fig. 12). When SiO_2f_/SiO_2_ after etching treatment, the brazed joint exhibited a 3D SiO_2f_/SiO_2_-metal gradient transition zone and the width of the zone increased with the etching depth increasing (see Fig. 11b–d). Further, the brazing alloy was able to fill up the space between the quartz fibers and formed a sound metallurgical bonding with them, due to the great wettability of E-SiO_2f_/SiO_2_. Furthermore, the “3D-pinning structure” can effectively reduce the residual stress in the joint, which decrease the continuous cracks and strengthen the joint. Thus, the shear strength of the joints increases from 5 MPa to 61.9 MPa with the etching depth increasing from 0 to 100 μm. However, the shear strength of the joints decreased with the etching depth increasing over 100 μm, because of the residual stress in the joints was increasing by the τ_xy_ of the “3D-pinning structure”, according to the FEA results (see Fig. 12).

**Figure 13:**
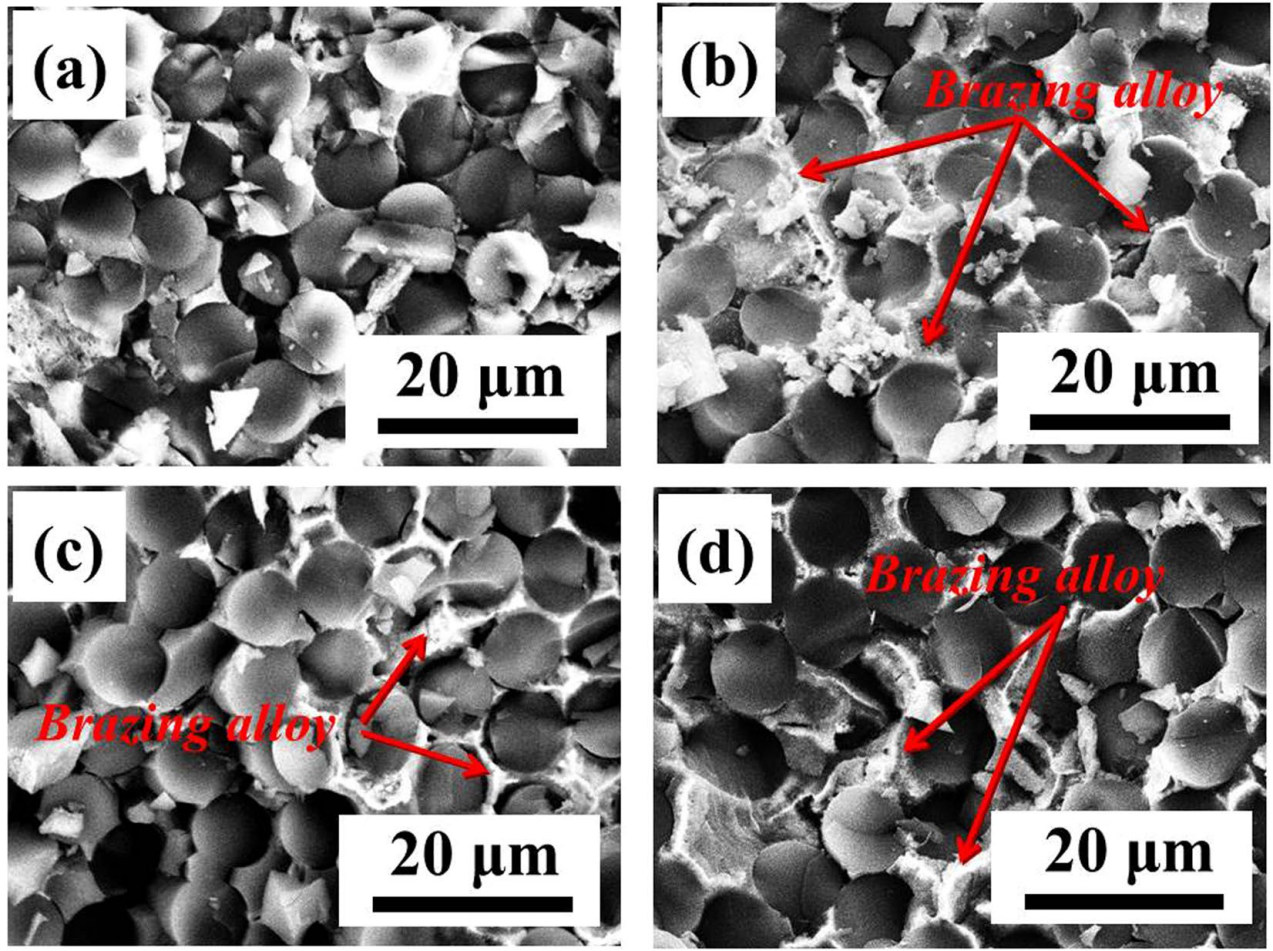
SEM images of the fracture surface in (**a**) 0 μm@E-SiO_2f_/SiO_2_-Nb, (**b**) 50 μm@E-SiO_2f_/SiO_2_-Nb, (**c**) 100 μm@E-SiO_2f_/SiO_2_-Nb and (**d**) 150 μm@E-SiO_2f_/SiO_2_-Nb joint.

**Figure 14:**
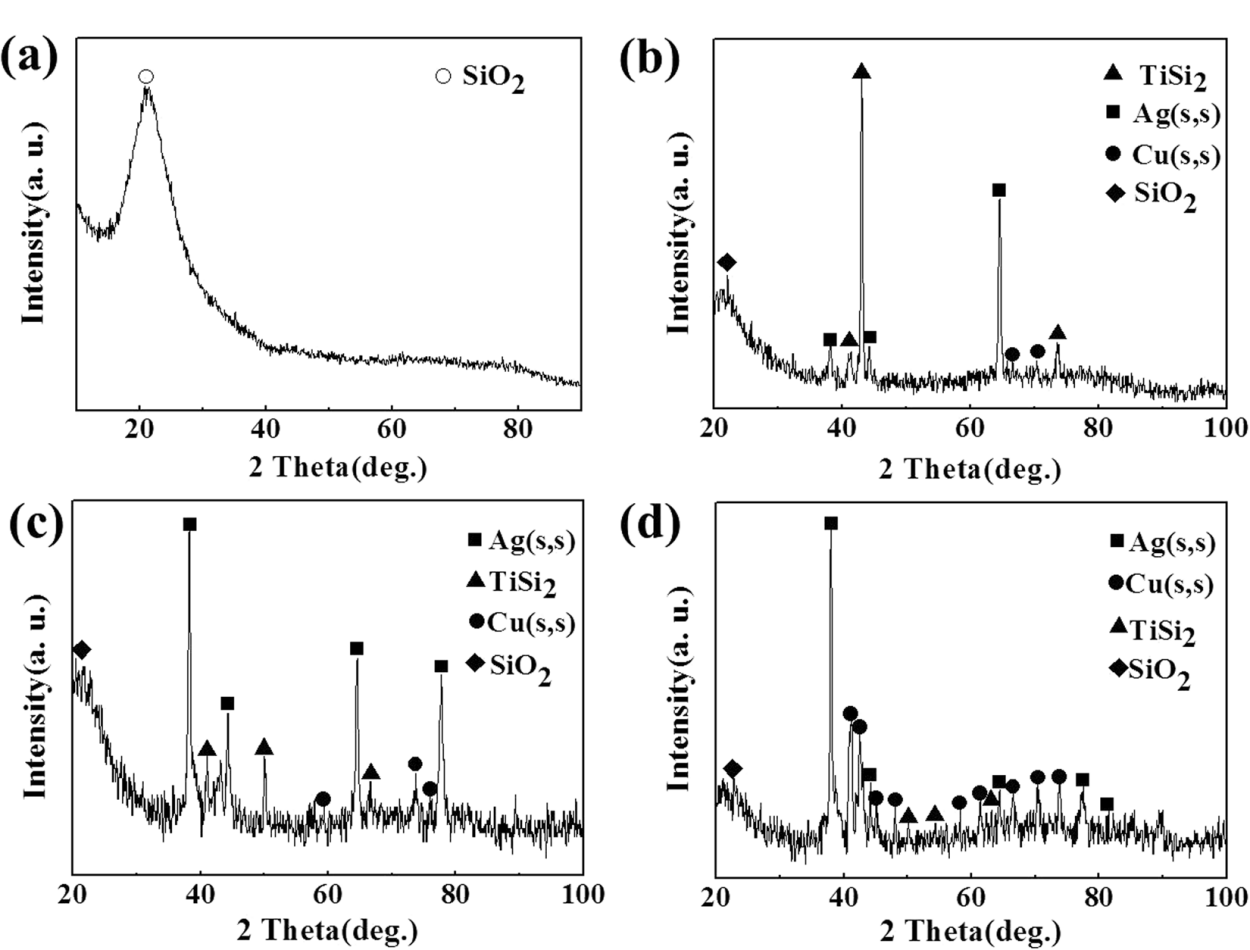
XRD result patterns of fracture surface of (**a**) 0 μm@E-SiO_2f_/SiO_2_-Nb, (**b**) 50 μm@E-SiO_2f_/SiO_2_-Nb, (**c**) 100 μm@E-SiO_2f_/SiO_2_-Nb, and (**d**) 150 μm@E-SiO_2f_/SiO_2_-Nb joint.

In order to further study the effect of the transition zone on the obtained joints, the fracture analysis was conducted. Figure 13 shows the fracture surface of joints after shear tests. It can be seen that only remained broken quartz fibers were left in the fracture of SiO_2f_/SiO_2_-Nb joint, as shows in Fig. 13a, which may be due to the cracks at SiO_2f_/SiO_2_ side caused by the high CTE mismatch. By contrast, almost the same type of fracture morphology for the E-SiO_2f_/SiO_2_-Nb joints can be observed in Fig. 13b–d. The brazing alloy infiltrated into E-SiO_2f_/SiO_2_ can be observed in fractures. In addition, XRD was used to confirm the reaction products at the fracture surface. As shown in Fig. 14a, it can be seen that the fracture of SiO_2f_/SiO_2_-Nb joint was only composed of amorphous silicon dioxide. Correspondingly, amorphous silicon dioxide, TiSi_2_, Ag(s,s) and Cu(s,s) were the reaction phases on the SiO_2f_/SiO_2_ composite fracture side for the fractures of 50 μm@E-SiO_2f_/SiO_2_-Nb, 100 μm@E-SiO_2f_/SiO_2_-Nb and 150 μm@E-SiO_2f_/SiO_2_-Nb joints (see Fig. 14b–d). According to the XRD patterns, it can be inferred that as for SiO_2f_/SiO_2_-Nb joint, fracture occurred along the brazing interface on the SiO_2f_/SiO_2_ composite side. It may be due to the residual stress constraining around the SiO_2f_/SiO_2_-AgCuTi interface. Moreover, for 50 μm@E-SiO_2f_/SiO_2_-Nb, 100 μm@E-SiO_2f_/SiO_2_-Nb and 150 μm@E-SiO_2f_/SiO_2_-Nb joints, fracture occurred in the 3D SiO_2f_/SiO_2_-metal gradient transition zone. Although the morphologies of the three kinds of fractures were almost the same, the mechanical properties of the joints were different, which may be owing to the residual stress concentrated in that zone.

Based on the above results, it is suggested that the FEA results can serve as a guide for the brazing test and a theoretical basis for the distribution of residual stress in the brazed joints. In addition, the results show clearly that the etching treatment plays a key role in two major aspects on active brazing SiO_2f_/SiO_2_ and Nb. On the one hand, it can effectively improve the wettability of SiO_2f_/SiO_2_, which is a precondition of successfully brazing SiO_2f_/SiO_2_ and Nb. On the other hand, it can induce the brazing alloy infiltrating into E-SiO_2f_/SiO_2_, which increases the bonded area, reduces the residual stress and enhances the mechanical properties. Therefore, regulating the surface structure is an easy and effective method to reduce the residual stress, which can strength the joints. As well the FEA and the brazing test results both reveal that the properties of 100 μm@SiO_2f_/SiO_2_-Nb joint is the best among the obtained brazed joints with different surface structures.

## Conclusions

In this paper, the optimized depth of brazing alloy infiltrating into SiO_2f_/SiO_2_ was achieved by combining FEA with experiments. After etching treatment, the fused silica with poor wettability has been consumed while the quartz fibers with good wettability were left, thus the wettability of E-SiO_2f_/SiO_2_ was improved. The good wettability of SiO_2f_/SiO_2_ played an important role in obtaining a high-quality joint. Moreover, “3D-pinning structure” formed in E-SiO_2f_/SiO_2_-Nb joints, which can reduce the residual stress in the joint by form the sound gradient transition of CTE. However, the residual stress rather reduced with the etching depth increasing over appropriate size, due to the τ_xy_ introduced by the “3D-pinning structure”. A relationship between the residual stress, the surface structure and the joint property in the brazed joints was demonstrated. As well the FEA and the brazing test results both realized the high-quality joint of E-SiO_2f_/SiO_2_-Nb joint and the shear strength of the joint reached 61.9 MPa, which was approximately 12 times than that of SiO_2f_/SiO_2_-Nb joint.

## Electronic supplementary material

Supplementary Material
