# Supplementary material for: The relation between residual stress, interfacial structure and the joint property in the SiO2f/SiO2-Nb joints
Source: Sci Rep. 2017 Jun 23;7:4187. doi: 10.1038/s41598-017-04531-w (PMC5482827; doi:10.1038/s41598-017-04531-w)
Supplement: Supplementary file 1 — Supplementary Material [file 41598_2017_4531_MOESM1_ESM.doc]

**Supplementary material**

**The relation between residual stress, interfacial structure and the joint property in the** **SiO_2f_/SiO_2_-Nb joints**

Qiang Ma, Zhuo Ran Li, Lai Shan Yang, Jing Huang Lin, Jin Ba, Ze Yu Wang, Jun Lei Qi*, Ji Cai Feng

*State Key Laboratory of Advanced Welding and Joining, Harbin Institute of Technology, Harbin 150001, China*

*Corresponding authors: Tel. /fax: 86-451-86418146;

E-mail: jlqi@hit.edu.cn (J L Qi)

**Designing of the model in FEA**

In this paper, the model utilized in FEA consisted of three pieces: SiO_2f_/SiO_2_ (or E-SiO_2f_/SiO_2_), AgCuTi brazing alloy and Nb (see Fig. 4a). It was worth noting that the special structure of the E-SiO_2f_/SiO_2_ by the etching treatment needed a completely new design system. Firstly, the E-SiO_2f_/SiO_2_ consisted of two pieces: etched (only the left braided quartz fibers) and no etched part, as shown in Fig. 4b. Next, it can be seen from Fig. 4c that AgCuTi brazing alloy was composed of infiltrating (the space between the left braided quartz fibers) and no infiltrating section. In this set up, the etched part of E-SiO_2f_/SiO_2_ and the infiltrating section of AgCuTi brazing alloy formed the “3D-pinning structure”. Finally, based on the microstructure of the E-SiO_2f_/SiO_2_-Nb joint, it can be designed that the proportion of the diameter of quartz fibers and the width of the space between each of the two quartz fibers was 3. To reduce simulation time and cost, the diameter of quartz fibers was set to 150 μm and only sixty-four quartz fibers in the form of the square (8 × 8) were analyzed (see Fig. 1c). In addition, for FEA, the depth of etched part was regulated from 0 to 150 μm and the width of the infiltrating section adjusted correspondingly while the width of the no infiltrating section kept 100 μm. And the physical and mechanical properties of materials applied in FEA were listed in Table S1.

Table S1 The physical and mechanical properties of materials applied in FEA [1]

| Materials | Temperature (^o^C) | Elastic modulus (GPa) | CET  (10^-6^K^-1^) | Yield stress (MPa) | Poisson’s ratio |
| --- | --- | --- | --- | --- | --- |
| SiO_2f_/SiO_2_ | - | 50 | 1.7 | 150 | 0.18 |
| AgCuTi | 20 | 93.6 | 19.0 | 330 | 0.35 |
|  | 200 | 85 | 19.7 | 297 |  |
|  | 400 | 79.4 | 20.2 | 225 |  |
|  | 600 | 70.2 | 20.5 | 52 |  |
|  | 800 | 58.1 | 21 | 20 |  |
|  | 2000 | 52.9 | 22.6 | 14 |  |
| Nb | 20 | 122 | 12 | 207 | 0.33 |
|  | 200 | 119 | 11 | 164 |  |
|  | 600 | 95 | 8.5 | 48 |  |
|  | 800 | 82.7 | 9.7 | 39 |  |
|  | 2000 | 75.8 | 11 | 31 |  |

**Reference**

[1] T.P. Wang, T. Ivas, W. Lee, C. Leinenbach, J. Zhang, Relief of the residual stresses in Si_3_N_4_/Invar joints by multi-layered braze structure – Experiments and simulation, Ceram. Inter. 42 (2016) 7080-7087.
